# A case of Gitelman syndrome with membranous nephropathy

**DOI:** 10.1186/s12882-022-02875-8

**Published:** 2022-07-26

**Authors:** Xiafei Guo, Shanshen Yu, Jia Sun, Lijun Mou

**Affiliations:** 1 Department of Nephrology, Linping Campus, The Second Affiliated Hospital of Zhejiang University School of Medicine, No.369, Yingbin Road, Linping District, 311199 Zhejiang Hangzhou, People’s Republic of China; 2 Division of Nephrology, Second Affiliated Hospital of Zhejiang University School of Medicine, No.88, Jiefang Road, Shangcheng District, Hangzhou, Zhejiang 310009 People’s Republic of China

**Keywords:** Gitelman syndrome, *SLC12A3*, Membranous nephropathy, Tacrolimus

## Abstract

**Background:**

Gitelman syndrome (GS) is a rare autosomal recessive inherited salt-losing tubulopathy (SLT). Here, we report, for the first time, a case of GS overlapping nephrotic syndrome (NS) related to PLA2R-associated membranous nephropathy (MN).

**Case presentation:**

We described a male patient had a 4-year history of recurrent fatigue. Serum biochemistry revealed hypokalemia with renal potassium wasting, hypomagnesemia, metabolic alkalosis, hyperreninemia, hypocalciuria, as well as nephrotic-range proteinuria, hypoalbuminemia, and elevated serum anti-phospholipase A2 receptor (PLA2R) antibody. Gene sequencing identified compound heterozygous mutations in *SLC12A3* [c.536T > A(p.V179D) and c.1456G > A(p.D486N)]. The unusual association of SLTs and nephrotic-range glomerular proteinuria prompted us to perform a renal biopsy. Renal biopsy showed idiopathic MN. Due to the potential to activate the sodium-chloride co-transporter (NCC) and cause hyperkalemia, tacrolimus was selected to treat NS. Following treatment with potassium chloride, magnesium oxide, low-dose glucocorticoid combined with tacrolimus, the fatigue significantly improved, and concurrently hypokalemia, hypomagnesemia were corrected and NS was remitted.

**Conclusions:**

Renal biopsy should be warranted for GS patients with moderate to nephrotic-range proteinuria. Tacrolimus was preferred to the management of GS patients with NS.

## Background

Gitelman syndrome (GS) is a rare autosomal recessive inherited salt-losing tubulopathy (SLT) because of the inactivating mutations in *SLC12A3* gene, which encodes the sodium-chloride cotransporter of distal convoluted tubules. GS is characterized by chronic hypokalemia, metabolic alkalosis, hypomagnesemia and hypocalciuria. GS typically is not classically associated with proteinuria, especially nephrotic-range glomerular proteinuria [1, 2]. Therefore, when nephrotic-range proteinuria developed in patient with GS, overlapping glomerular diseases should be considered. PLA2R-associated membranous nephropathy (MN) is an auto-immune disease characterized by moderate to nephrotic-range proteinuria, a common cause of nephrotic syndrome (NS) in adults [3, 4]. Up to now, GS with concomitant MN has not been reported. We described one patient with GS overlapping MN. He was successfully treated with tacrolimus and glucocorticoid, potassium and magnesium supplementation.

## Case presentation

A 24-year-old male patient was admitted to our hospital on Sep 1^st^, 2020 due to recurrent limb fatigue for four years and aggravating for one month. Four years ago, the patient presented limb weakness episode once sweating a lot in every summer, hypokalemia was revealed in a local hospital. Following potassium chloride supplement during hospitalization, fatigue was relieved. However, he stopped taking potassium chloride after discharge, and fatigue recurred following sweating in every summer.

One month ago, the patient suffered from limb weakness again after tooth extraction. Serum biochemistry in local hospital showed that potassium was 2.8mmol/L and albumin was 22 g/L; urinalysis demonstrated 3 + proteinuria. Therefore, he was admitted to our hospital. The patient denied taking diuretics, laxatives, diet pills and Chinese herbal medicines; His parents are healthy and non-consanguineous marriage; his two sisters are healthy.

**Fig. 1:**
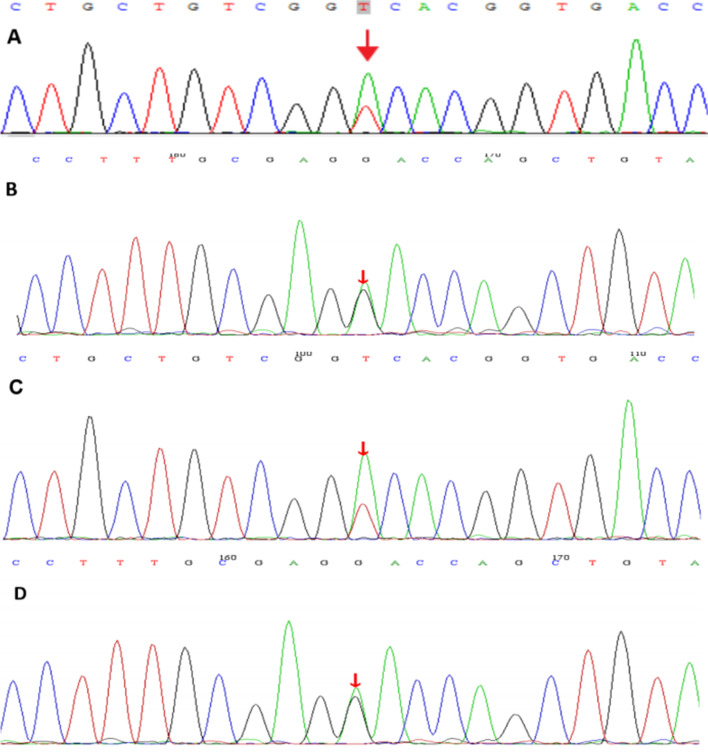
Gene analysis results. **A**, **C**. The patient and his father carry a c.536T>A, p.V179D (arrow) heterozygous mutation in *SLC12A3 *gene, respectively and his mother has no mutation in *SLC12A3 *gene at this site. **B**, **D**. The patient and his mother carry a c.1456G>A, p.D486N (arrow) heterozygous mutation in *SLC12A3* gene, respectively and his father had no mutation in* SLC12A3 *gene at this site

Blood pressure after admission was 108/77mmHg.While this patient had no oedema and other physical examination abnormality. Laboratory tests showed hypokalemia due to renal potassium wasting, hypomagnesemia, hypochloridemia, metabolic alkalosis, hyperreninemia, hypocalciuria (Table 1), which were suggestive of GS. Thiazide test was performed according to the protocol described in the previous study [5]. The difference value between the chloride excretion fraction before and after the use of hydrochlorothiazide was 0.468%, indicating that he was no response to hydrochlorothiazide, which could be functionally diagnosed of GS. Therefore, Next-Generation sequencing(NGS)-based panel was performed to identify the exact type of SLTs. The method of gene sequencing was performed as previously reported [6]. Two missense heterozygous mutations[c.536T > A(p.V179D) and c.1456G > A(p.D486N)] in *SLC12A3* were revealed. His father carries one mutation (c.536T > A) and his mother carries the other mutation (c.1456G > A), which are compatible with compound heterozygosity in this patient (Fig. 1). The mutation of *SLC12A3* ( p.V179D) was predicted to be benign by PolyPhen-2, polymorphism by Mutation Taster, and damaging by SIFT. This mutation has been reported by Lee, et al. [7]. The mutation of p.D486N was predicted to be probably-damaging by PolyPhen-2, disease-causing by Mutation Taster, and damaging by SIFT. This mutation has been reported by Simon, *et a*l [8]. Both mutations are classified as likely pathogenic according to American College of Medical Genetics(ACMG) guidelines. Eventually, GS was identified.

**Table 1:** Comparison of patients before and after treatment in laboratory tests

Examination item	2021.09.02	2021.03.07	Reference value
Serum biochemistry			
potassium(mmol/L)	3.00	4.09	3.50 ~ 5.50
magnesium(mmol/L)	0.57	0.65	0.73 ~ 1.06
calcium(mmol/L)	2.01	2.48	2.08 ~ 2.60
sodium(mmol/L)	144.8	143.3	135.0 ~ 145.0
chloride (mmol/L)	101.9	101.5	96.0 ~ 106.0
creatinine (µmol/L)	63	93	40 ~ 106
albumin (g/L)	24.5	44.9	35.0 ~ 52.0
cholesterol(mmol/L)	5.21	5.47	3.0 ~ 5.7
triglycerides(mmol/L)	4.70	1.86	< 1.7
PLA2R (RU/mL)	25.4	2.1	< 20.0
Upright plasma renin (uIU/mL)	294.1	-	4.4 ~ 46.1
Upright plasma aldosterone (pg/mL)	108.0	-	30.0 ~ 353.0
Arterial blood gas analysis			
PH value	7.442	-	7.350 ~ 7.450
Base excess (mmol/L)	6.3	-	-3.0 ~ 3.0
HCO3^−^ (mmol/L)	30.9	-	22.0 ~ 26.0
PaCO2 (mmHg)	46	-	36.0 ~ 44.0
Urine analysis			
proteinuria (g/L)	2+	1+	negative
hematuria (number/ul)	5	< 1	< 12
Albumin to creatinine ratio (mg/g.Cr)	3083.3	356.3	< 25.0
24 h proteinuria (mg/24 h)	4838.4	-	22.0 ~ 132.0
24 h potassium (mmol/24 h)	57.60	-	25 ~ 100
Spot urinay calcium/creatinine (mmol/mmol)	0.022	-	-
Spot urinay potassium /creatinine (mmol/mmol)	8	-	-

**Fig. 2:**
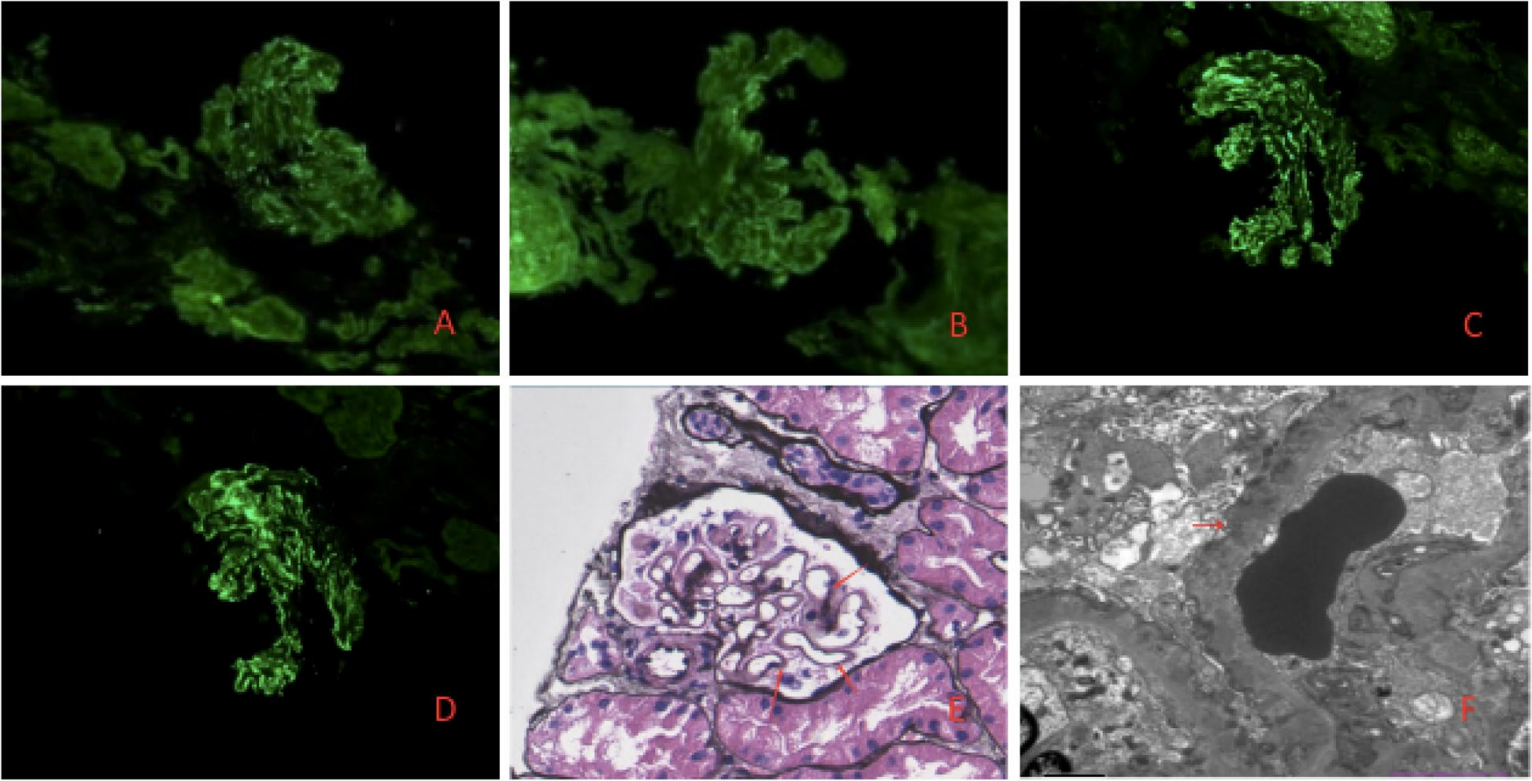
Renal pathology. **A**, **B**, **C **and **D**: PLA2R、IgG、IgG1、IgG4 were granular deposition along capillaries (Immunofluorescence×200, fluorescence microscope is OLYMPUSmicroscope), respectively; **E**: spike like structure (red arrow), and subepithelial deposition of erythrophilic proteins (PASM×400, light microscope is OLYMPUSmicroscope); **F**: Electron microscope showed subepithelial deposition of electron dense substance (red arrow), diffuse foot process effacement (Electron microscope×5000, electron microscope is JEOL)

24 h proteinuria showed nephrotic-range proteinuria, he also has hypoalbuminemia. Results of ANA, ENA, anti-dsDNA, anti-tiroid antibodies, imaging of chest/abdomen were negative. Taken together, nephrotic syndrome (NS) was identified. Therefore, renal biopsy was performed. Light microscopy showed 18 glomeruli, including 3 sclerotic ones, with thickened basement membrane, spike-like structures and subepithelial deposition of erythrophilic proteins.; The immunofluorescence results of renal biopsy showed that IgG (3+), IgG1 (3+), IgG4 (3+), C3 (+) and PLA2R (2+) were granular deposition along capillaries; Electron microscopy showed irregular thickening of glomerular basement membrane with the thickest part of 1500 nm, diffuse foot process effacement, and deposition of electron dense substance in subepithelial and basement membrane. The serum anti-phospholipase A2 receptor (PLA2R) antibody was also positive. Eventually, diagnosis of PLA2R-associated MN was established (Fig. 2).

According to the patient’s history, laboratory tests and gene sequencing findings, the final diagnosis was GS concomitant with MN. Following immunosuppressant with methylprednisolone tablets (12 mg once daily) plus tacrolimus (1 mg twice daily), electric supplement with potassium chloride tablets (3.0 g per day), magnesium oxide (300 mg magnesium per day), the level of serum potassium and magnesium increased, NS was partially remitted, anti-PLA2R titre reduced after 6 months (Table 1).

## Discussion and conclusions

GS is a rare autosomal recessive inherited SLT. American doctor Gitelman first reported 3 cases of familial hypokalemia, hypomagnesemia, hypocalciuria and metabolic alkalosis in 1966. The cause of GS is the inactivating mutations of *SLC12A3* gene, which encodes the sodium-chloride co-transporter of distal convoluted tubules [8]. GS are characterized by hypokalemia, hypomagnesemia, metabolic alkalosis, hypocalciuria, secondary renin-angiotensin-aldosterone (RAAS) activation, normal or low blood pressure [9]. GS patients may manifest fatigue, salt craving, polydipsia, polyuria, and paroxysmal tetany triggered by hypomagnesemia [10]. It is suggested GS patient intake potassium and magnesium supplements and sodium chloride-containing food for life [11], as GS is caused by the deficiency of sodium-chloride co-transporter.

However, nephrotic-range glomerular proteinuria in GS patients is rare generally. At present, there are 6 cases of GS accompanied by moderate to nephrotic-range proteinuria [12–17]. Two of the patients were female and four were male; Three patients presented with nephrotic syndrome, two of whom received glucocorticoid therapy; 5 patients underwent renal biopsy, including C1q nephropathy, minimal change disease (MCD), diabetic nephropathy and 2 cases of focal segmental glomerulosclerosis (FSGS) (Table 2).

**Table 2:** Basic information of 6 cases

Case	Sex	Age	Proteinuria	Serum albumin	Edema	Renal pathology	Use of glucocorticoids
Hanevold C, et al. [13]	Female	12	2.5 mg/mg	3.5 gm/dL	No	C1qN	Not used
Pandey DB, et al. [14]	Female	19	1.445gm/d	2.6 gm/dL	Yes	N/A	Glucocorticoid
Ceri M, et al. [15]	Male	32	1094 mg/d	N/A	NO	FSGS	Not used
Demoulin N, et al. [16]	Male	27	770 mg/d	N/A	N/A	FSGS	N/A
Chen Q, et al. [17]	Male	47	10.20 g/d	22 g/L	N/A	MCD	Glucocorticoid
Chen Q, et al. [12]	Male	40	2793 mg/d	N/A	N/A	DN	Not used

*N/A *Not applicable, *C1qN* C1q Nephropathy, *FSGS* Focal segmental glomerulosclerosis, *MCD* Minimal change disease, *DN* Diabetes nephropathy

Therefore, renal biopsy should be warranted for GS patients with moderate to nephrotic-range glomerular proteinuria. If there are primary glomerular diseases, glucocorticoid or other drugs may be added, in addition to potassium and magnesium supplements for GS [12, 17].

So is there a correlation between GS and glomerular proteinuria? It has been reported that the possible mechanisms between GS and glomerular proteinuria is the chronic activation of RAAS, leading to increased systemic and local levels of angiotensin-II and renin, may in turn cause podocyte lesions. Angiotensin II induced proteinuria through vascular endothelial growth factor and transforming growth factor-β1 (TGF-β1) [12, 16]. At present, abnormal heterogeneity of basement membrane thickness and disappearance of podocyte foot processes, as well as decreased expressions of nephrin and podocin, have been observed in transgenic mice overexpressing renin. TGF-β1 is considered the major pro-fibrotic agent in renal disease [18]. Nephrotic range proteinuria was also present in patients with Addison’s disease, a disease associated with hyperreninemia, and renal biopsy showed FSGS, nodular deposition of IgM, and C3 [19]. Severe intraglomerular detachment of podocytes was described in another case of GS before, which was also associated with decreased renal nephrin expression [20]. Chronic hypokalemia may also cause proteinuria, Reungjui et al. detected mild proteinuria in hypokalemic rat models with or without hydrochlorothiazide. For the same degree of hypokalemia, renal injury was more obvious in the hydrochlorothiazide treated group, which was attributed to the secondary hyperaldosteronism due to long-term volume depletion [21]. According to the above studies, there is similar RAAS activation caused by blood volume depletion in GS patients. Therefore, there may be a correlation between GS and FSGS.

PLA2R-associated MN is an auto-immune disease, characterized by non-inflammation mediated subepithelial immune complex deposition with diffuse thickening of glomerular basement membrane. Approximately 70-80% of patients with primary MN have circulating PLA2R antibodies [22]. Renal pathology of this patient we described was MN, with increased titer of serum PLA2R antibody. Therefore, it was an immune related disease rather than a metabolic related disease. By above knowable, we consider that FSGS may be related to the pathophysiological changes of GS in GS patients with nephrotic-rangeproteinuria, while other pathological changes may be irrelevant to GS, including PLA2R-associated MN, which involves an autoimmune response. In addition, GS is commonly diagnosed in children and young adults, such as the patient described, MN is untypical at the age of the patient, which still suggest the association between GS and PLA2R-associated MN is probably coincidental. At this time, renal biopsy is supposed to be necessary.

In the treatment, we chose a low-dose glucocorticoid with tacrolimus and supplements of potassium chloride and magnesium oxide. 2017 Kidney Disease: Improving Global Results (KDIGO) guidelines recommended that the initial dose of potassium chloride supplement for adults should be 40 mmol (equivalent to 3.0 g) per day, and suggests that the serum potassium and magnesium levels of GS patients should be maintained at 3.0 mmol/L and 0.6 mmol/L at least, respectively [23]. We gave the patient an initial dose of 3.0 g potassium chloride per day, the serum potassium easily increased to 4.09mmol/L consequently. We found that serum potassium of the patient exceeded the target value of KDIGO, and the proteinuria was effectively controlled through treatment. It has been reported that tacrolimus has the potential to increase the activity of phosphorylated NCC and NCC regulated kinases WNK3, WNK4 and SPAK, resulting in over activation of NCC, leading to hyperkalemia, similar to Gordon syndrome or familial hyperkalemic hypertension [24, 25]. This may explain why the patient’s serum potassium is easy to surpass the standard by far.

In conclusion, we report a case of GS concomitant with MN. Renal biopsy should be warranted for GS patients with moderate to nephrotic-range glomerular proteinuria in order to guide treatment. In addition, due to the potential to activate NCC and cause hyperkalemia, tacrolimus may have more advantages in the treatment of GS patients with NS secondary to PLA2R-associated MN.

## Data Availability

Data regarding this study were obtained from clinical charts stored in the physician office records of Second Affiliated Hospital of Zhejiang University School of Medicine, therefore, cannot be shared. Any reasonable request to access the data must be approved before the data can be released. The datasets used and/or analyzed during the current study are available from the corresponding author on reasonable request.

## Abbreviations

GS: Gitelman syndrome
SLT: salt-losing tubulopathy
NS: nephrotic syndrome
MN: membranous nephropathy
PLA2R: anti-phospholipase A2 receptor
NCC: sodium-chloride co-transporter
NGS: next-generation sequencing
ACMG: American College of Medical Genetics
RAAS: renin-angiotensin-aldosterone
MCD: minimal change disease
FSGS: focal segmental glomerulosclerosis
TGF-β1: transforming growth factor-β1
KDIGO: Kidney Disease:Improving Global Results
